# Luminescence sediment tracing reveals the complex dynamics of colluvial wedge formation

**DOI:** 10.1126/sciadv.abo0747

**Published:** 2022-06-01

**Authors:** Harrison Gray, Christopher DuRoss, Sylvia Nicovich, Ryan Gold

**Affiliations:** 1 U.S. Geological Survey, Geosciences and Environmental Change Science Center, Denver, CO 80225, USA.; 2 U.S. Geological Survey, Natural Hazards Science Center, Golden, CO 80401, USA.; 3 Bureau of Reclamation, Technical Services Center, Denver, CO 80225, USA.

## Abstract

Paleoearthquake studies that inform seismic hazard rely on assumptions of sediment transport that remain largely untested. Here, we test a widespread conceptual model and a new numerical model on the formation of colluvial wedges, a key deposit used to constrain the timing of paleoearthquakes. We perform this test by applying luminescence, a sunlight-sensitive sediment tracer, at a field site displaying classic colluvial wedge morphostratigraphy. The model and data comparison reveals complex sediment transport processes beyond the predictions of either conceptual or numerical models, including periods of simultaneous debris and wash facies forming processes, erosion, and reworking. These processes could lead to preservation bias, such as incomplete or overinterpretable paleoearthquake records, given the right environmental conditions. Attention to the site-specific mechanics of fault zone depositional systems, such as via sediment tracing, may buffer against the possible effects of preservation bias on paleoseismic study.

## Teaser

Creative application of contemporary light emission data identifies complexities of prehistoric earthquake records.

## INTRODUCTION

Reconstructing prehistoric earthquake events from fault zone sedimentary deposits is a fundamental goal of paleoseismology. However, understanding how such deposits may or may not record a complete tectonic history remains an outstanding challenge with implications toward our understanding of seismic hazard and earthquake mechanics (*1*, *2*). Solving this challenge requires that we continually examine and test our conceptual frameworks of fault zone evolution.

**Fig. 1.**
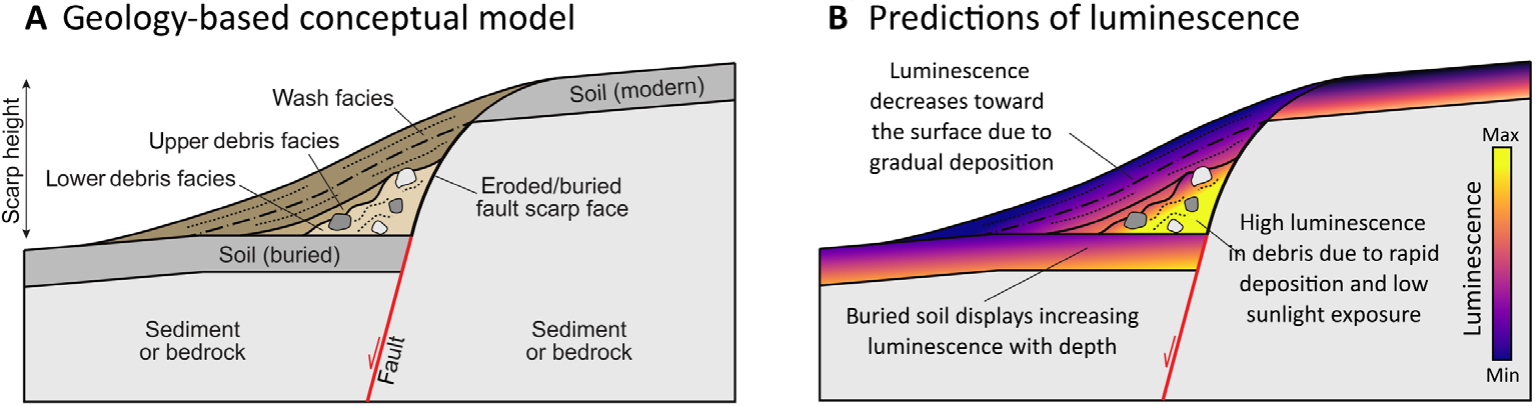
Predictions of the conceptual model. (**A**) An idealized schematic of the colluvial wedge conceptual model. In the model, a colluvial wedge will demonstrate various sedimentary facies, resulting from the collapse of the fault scarp and progressive colluvial sediment transport. Colors are to highlight contrast between units and do not represent facies characteristics or provenance. (**B**) The conceptual model relies on interpretations on the mechanics of sediment transport. From these interpretations, one can infer a predicted spatial distribution of luminescence across an idealized colluvial wedge. Note that this conceptualization represents a simplified endmember. Various scenarios of depositional processes could create more complicated patterns of luminescence.

The colluvial wedge model (Fig. 1A) is a widespread conceptual framework for interpreting the wedge-shaped sequences of colluvium that collect at the base of, typically normal motion, fault scarps (*3*). This conceptual model is heuristically developed from field observations of the sedimentology of ancient and historic colluvial wedges (*1*, *3*, *4*). A typical application of the conceptual model involves an excavation of a fault zone, classification of the exposed sedimentary deposits, and then interpretation of the number of earthquake events represented from the stratigraphic and structural relationships. The timing of these relationships is constrained with geochronological data, often radiocarbon and luminescence ages, to support models of earthquake timing and recurrence. These models then feed seismic hazard assessments that quantify the frequency and magnitude of shaking resulting from major earthquakes. Such assessments inform decision-making and policy at local, state, and federal levels. Through this pipeline, the foundational colluvial wedge model has notable societal impacts on policymaking and potentially the lives and livelihoods of millions.

Despite this importance, studies validating the conceptual model are largely absent beyond the routine application of it in paleoseismic study. In particular, there appear to be no studies focused on the seconds to thousand-year time scales over which colluvial wedge stratigraphy develops. Studies on the sediment dynamics of modern fault scarps and colluvial wedges have worked to fill this gap as best as possible (*5*). However, it is worth noting that the environmental conditions of the present may not be representative for all past fault scarps. We aim to complement previous research by performing a validation of the colluvial wedge conceptual model with new luminescence sediment tracing and numerical methods that reveal colluvial wedge dynamics over the long time scales of colluvial wedge formation.

To validate the conceptual model, we trace the paths of sediment across a colluvial wedge using luminescence, a form of trapped-charge phenomena wherein crystallographic defects in mineral sand grains allow for the trapping of locations of positive charge (holes) and negative charge (electrons) in meta-stable energy states (*6*). When buried, mineral sand grains accumulate charge in defects owing to background ionizing radiation. Conversely, when a mineral grain is exposed to sunlight, sunlight photons allow electrons to transition across energy states and allows for recombination between electrons and holes (*6*). In geomorphic environments, luminescence can act as a sediment tracer as the amount of sunlight exposure versus burial time varies, depending on geomorphic processes and their rates (*7*). Here, we validate the colluvial wedge conceptual model and its foundational sediment transport dynamics by applying this sediment tracer concept, via numerical modeling and a new application of portable optically stimulated luminescence (portable OSL), to a colluvial wedge with classic morphostratigraphy.

### The Deep Creek natural exposure

**Fig. 2.**
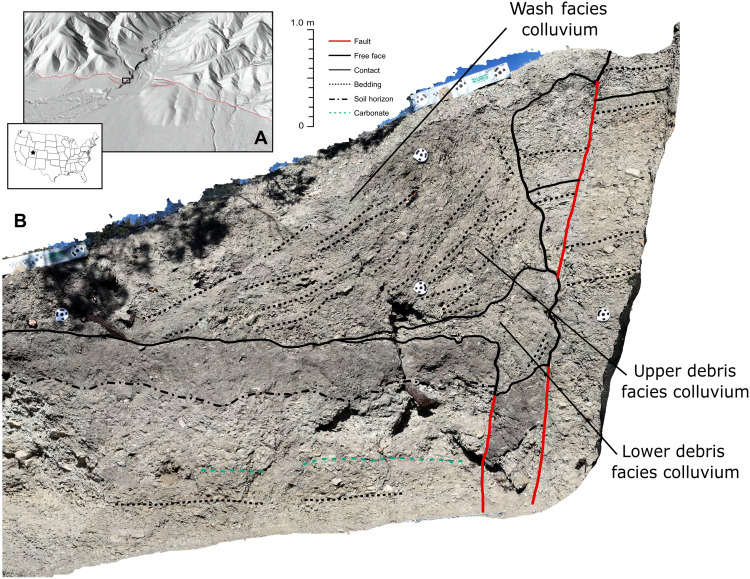
Introduction of the Deep Creek natural exposure. (**A**) Location and setting of the field site. Image is a 0.5 m LiDAR (Light Detection and Ranging) digital elevation model obtained from opentopography.org. Location is at 39.507574°, −111.861834°. Red line indicates mapped trace of the Wasatch Fault. (**B**) Orthophoto of the Deep Creek natural exposure with interpreted contacts from the study of DuRoss *et al.* (*11*). Unannotated orthophoto reproduced as fig. S8.

The Deep Creek natural exposure (39.5075°, −111.8618°) is a colluvial wedge resulting from a 2-m tall scarp produced by a normal-faulting earthquake on the Levan segment of the Wasatch Fault, Utah, USA (*8*, *9*). The earthquake had a likely magnitude of 6.9 ± 0.2 at about 500 to 700 years ago (*10*–*12*). The Deep Creek exposure exhibits classic colluvial wedge stratigraphy as predicted by the colluvial wedge conceptual model. The exposure consists of basal poorly-to-weakly sorted sedimentary “lower debris” facies overlain by similarly sized yet bedded “upper debris” facies, which are capped by finer-grained and better sorted “wash” facies (Fig. 2) (*11*). From similar observations of facies in other colluvial wedges, the conceptual model posits that sediment deposition occurs by two sequential phases: high-energy gravitational collapse of the fault scarp followed by gradual deposition by various lower-energy sediment transport and soil formation processes (*1*, *4*, *5*). An application of the conceptual model to the Deep Creek natural exposure, based on the sedimentology alone, results in a straightforward interpretation: the lower and upper debris facies deposited immediately during or after the earthquake followed by gradual deposition of the overlying wash facies up to the present.

**Fig. 3.**
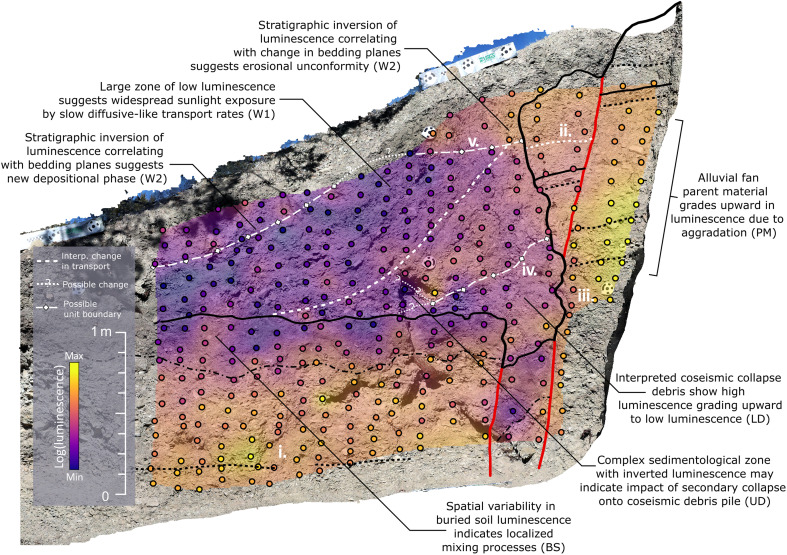
Deep Creek natural exposure with portable luminescence measurements (filled circles) and overlaid interpolated luminescence map with interpreted contacts representing changes in sediment transport given by white lines. Annotations describe interpretations of sediment transport mechanics from luminescence using principles described in the main text. Symbols in parentheses refer to units in Table 1. Roman numerals refer to locations discussed in the main text.

To investigate the spatial distribution of luminescence, we use the use of a portable luminescence reader manufactured by SUERC (Scottish Universities Environmental Research Centre) (*13*). A portable luminescence reader consists of a sample chamber, blue and infrared light-emitting diodes, and photomultiplier tube. The device performs measurements by counting the photons emitted under illumination of aliquots of sediment with little to no pretreatment. Here, we sieve each sample to the 90- to 250-μm grain size and weigh each sample to 0.5 ± 0.005 g to maximize the comparability of aliquots (*14*). We collected and measured 342 samples with an approximately 10 cm–by–15 cm grid spacing across the 3 m–by–2 m outcrop without regard for stratigraphic contacts (Fig. 3) (*11*). We then interpolate the blue stimulated luminescence measurements into a two-dimensional map using an inverse distance weighing function with a cell size of 10 cm and a power of 1 to show the broad patterns in the data (Fig. 3). Note that the interpolation is used primarily for illustration, and an analysis of interpolation methods and kernel size is given by DuRoss *et al.* (*11*). We show the natural logarithm of the photon counts to control for the orders of magnitude variance in apparent sensitivity. Last, we note that the dose rate across the exposure is an approximately uniform 1.99 ± 0.16 gray/ka, and the ratio of infrared stimulated luminescence to blue stimulated luminescence is approximately constant across the wedge and alluvial fan parent material, suggesting a uniform lithologic composition (*11*).

## RESULTS

The portable luminescence map reveals a rich and complex stratigraphy beyond that observable with sedimentology alone. First, the data show a general decrease in luminescence with horizontal distance from the fault and with vertical distance from the underlying buried soil (Fig. 3). The gradient in both directions is not monotonic, with notable variance and layering of areas of high and low luminescence in all sedimentary facies. Locations of higher luminescence appear as both wedge-shaped features and as limited bright locations throughout the exposure. Notably, the lower, presumably collapse-related, debris facies have lower luminescence than the nearby alluvial fan parent material and the overlying upper debris facies and have similar values to the contacting buried soil (Fig. 3). The upper, better sorted, debris facies show a prominent wedge-shaped zone of higher luminescence, which grades toward a wide region of lower luminescence with distance from the fault zone (Fig. 3). Near the surface, the luminescence is notably higher than stratigraphically lower levels for both the fault-proximal and fault-distal regions.

**Fig. 4.**
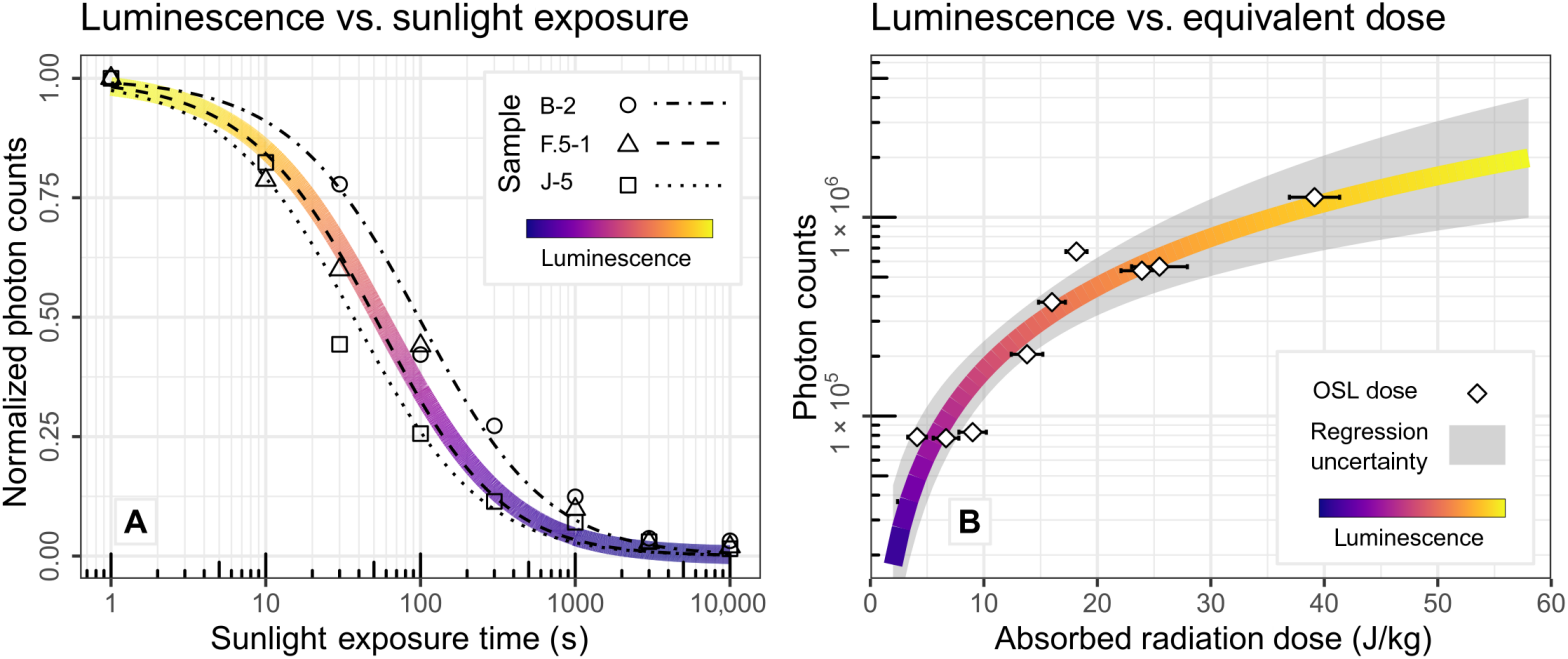
Data from portable luminescence experiments used to interpret luminescence tracer data (Fig. 2) and to produce numerical predictions (Fig. 5). (**A**) Results of exposing aliquots of three samples to direct sunlight for discrete time intervals. Measurements are normalized by the nonsunlight-exposed value for use in the numerical model. Color line shows fitted general-order kinetic equation applied to all three samples (see Materials and Methods for equations and values) (*24*–*26*). Black lines show fit to individual samples. Initial luminescence is reduced to >3% after 50 min. (**B**) Linear relationship between portable luminescence measurements and equivalent dose (*25*) measured using standard multigrain OSL dating on quartz (*15*). Both color scales are matched to Fig. 3. Regression uncertainty is the 95% confidence interval.

We used a combination of the patterns of luminescence and sedimentary characteristics to map and interpret changes in either aggradation or transport mechanics (Fig. 3). The guiding principles to produce this map are developed from previous concepts of luminescence as a sediment tracer (*7*) and from a sunlight exposure experiment to quantify the time scales of luminescence removal (Fig. 4). These principles are the following: (i) Relative differences in luminescence represent a change in the proportion of sunlight exposed to nonsunlight-exposed grains; (ii) changes in the proportions of sunlight exposure are a function of grain transport mechanics, including velocity and provenance; (iii) stratigraphic inversions where high luminescence overlies low luminescence, over more than one sample, indicate a change in mechanics such as rapid deposition or a change in sediment source location from the scarp; and (iv) luminescence inversions that accompany a change in sedimentary texture, such as clast orientation, potentially indicate interpretable boundaries in transport mechanics. From these principles, we annotate our interpretations of development at Deep Creek in Fig. 3. These interpretations serve as the basis for estimating depositional time scales and comparing model predictions as described below.

Note that natural variability of the portable luminescence can introduce nuance to interpretations of spatial patterns. Some features are present in the data that are nuanced when compared to the principles above but do not alter the main conclusions of the study. The first is that there can be lateral variability in portable luminescence within intact stratigraphy (location i in Fig. 3). These variations may relate to subtle changes in mineralogy even within the uniform provenance of the alluvial fan parent material. Another nuance is the vertical pattern of luminescence within a down-dropped section of stratigraphy immediately adjacent to the fault that shows a stratigraphic inversion in luminescence despite being within apparently intact stratigraphy (location ii in Fig. 3). This luminescence inversion is coincident with a pebble-sized, well-sorted, and clast-supported layer. A possible explanation for this inversion is that the layer in question represents rapid or turbid deposition, such as a debris flow, that is preserved in the alluvial fan parent material. Unfortunately, the extent of our data does not include the apparently correlating layer on the other side of the fault to confirm this hypothesis. A final nuanced feature is the measurements made immediately to the right of the mapped fault (location iii in Fig. 3). These measurements are not laterally consistent with other the measurements made within the footwall. We invoke two possibilities: (i) Faulting within the alluvial fan deposits has translocated material along the fault, and/or (ii) the erosional contact between the colluvial wedge and alluvial fan (heavy line; Fig. 3) represents a gradational zone of sediment mixing. Both processes could create a portable OSL discrepancy that was not observable in the coarse-grained units of the parent material.

### Time scales and stochasticity of wedge-forming processes

The luminescence tracer data outline depositional processes where we can use geochronology to infer the apparent time scales over which these processes occur. To do this, we import data from prior C14 and traditional OSL geochronology (*15*) and paleoseismology (*11*) to develop a depositional sequence (Table 1). We first must consider what these geochronometers represent in a colluvial wedge environment. The C14 and OSL methods date plant death and previous sunlight exposure for detrital or bulk microscopic organic material and fine quartz sand, respectively. The time since plant death includes the time since colluvial burial, the time of transport before burial, and potentially any time spent in a prior burial period. Burial time is only represented by luminescence if sunlight exposure before burial is sufficient to remove all luminescence; otherwise, some amount of inherited time will be included. In a fault scarp environment, rapid collapse of the free face has a high probability of recycling carbon from the parent material and a potentially low probability of sunlight exposure. Conversely, slower depositional processes may have a higher probability of incorporating contemporary carbon and inducing extensive sunlight exposure. Note that the traditional OSL ages use methods involving quartz OSL that resets far faster in sunlight than the portable luminescence and statistical methods biased toward the most sunlight-exposed grains. These methods produce agreement with neighboring C14 ages.

**Table 1.** Geochronologic constraints on depositional mechanics interpreted from portable luminescence data of the Deep Creek natural exposure (Fig. 3). Geochronologic ages are from the study of Gray *et al.* (*15*) and earthquake timing results from Bayesian analysis by DuRoss *et al.* (*11*). Apparent process time scale represents the approximate order of magnitudes represented by the geochronologic bounds (minimum age/maximum age). Letters in parentheses represent units marked on Fig. 3. Max, maximum; Min, minimum.

**Event**	**Max age**	**Min age**	**Process type**	**Apparent process time** **scale**
	**(ka)**	**(ka)**		
Deposition of parent material alluvial fan (PM)	> 13.1 ± 1.1	4.8 ± 0.2	Alluvial deposition	Thousands of years
Abandonment of parent material alluvial fan	4.8 ± 0.2	Ongoing	Fluvial incision	Approximately thousands of years
Development of hanging wall soil (BS)	3.7 ± 0.4	0.13 ± 0.01	Soil development	Tens to thousands of years
Earthquake event	0.68 ± 0.18	0.41 ± 0.04	Scarp formation	Seconds
Formation of lower debris facies (LD)	0.68 ± 0.18	0.32 ± 0.04	Coseismic deposition plus postdepositional vmodification	Tens to hundreds of years or less
Formation of upper debris facies (UD)	0.32 ± 0.04	0.17 ± 0.01	Stochastic rapid deposition	Hundreds of years
Formation of wash facies (W1)	0.32 ± 0.04	0.17 ± 0.01	Gradual slow deposition	Hundreds of years
Formation of nearest surface unit and modern soil (W2)	0.17 ± 0.01	Ongoing	Potential erosion and/or stochastic rapid deposition	Tens of years or more

We extract time scales for the dominant sedimentary processes acting across the Deep Creek exposure using the C14 and OSL geochronology. We assume that the oldest ages within colluvial wedge units have a high probability of overestimation due to carbon recycling and incomplete sunlight exposure. Thus, we use the minimum ages of each unit to constrain timing where possible. In one case, the lower debris facies lack the geochronology to constrain timing, so we use a single portable OSL–derived age to fill this data gap. To avoid circularity, we only use a single portable OSL age where the mapping of units does not depend on this sole data point (Fig. 3). Next, we assume that the representative process time scale is defined by the order(s) of magnitude as the range represented by the geochronology. A unit spanning hundreds of years from depositional start to end is interpreted to represent a process that takes hundreds of years to occur.

Note that by stating an order of magnitude range as representative, we are bracketing the complete suite of potential stochastic subevents and/or hiatuses that characterize the process. For example, a collapse may occur in seconds, but there may be years to hundreds of years between collapses or other transport processes operating to produce the sedimentary unit. These time scales give us a broad sense of the stochastic nature of deposition within a colluvial wedge. We also caution that our field site is a two-dimensional exposure of a three-dimensional feature, and thus, an order of magnitude is a conservative estimate given data limitations. We deem these process time scales as apparent values for post-earthquake event processes to acknowledge that uncovering the full time scale range may require future study beyond our single field site.

Combining the geochronology with our luminescence tracer–delineated units results in a series of apparent time scales of wedge-forming processes. The apparent process time scales range over seconds to hundreds of years. The most notable observation is the overlap in time scales between debris facies, wash facies, and soil-forming processes (Table 1). This overlap suggests that both wash and debris facies can result from 100-year apparent process time scales. Soil-forming processes at this field site appear to span a wide range inclusive of the 100-year time scale of wash and debris colluviation. Processes such as deposition and abandonment of the alluvial fan parent material appear to span longer thousand-year time scales and are not directly involved in the formation or modification of the colluvial units. Comparison of these time scales with the models and the resulting implications are given in the following sections.

## DISCUSSION

### Conceptual and numerical predictions

We developed predictions of spatially variable patterns in luminescence from the conceptual model and from cellular automata numerical modeling (*16*). For the conceptual model predictions, either we use literature on colluvial wedge sedimentation, literature on luminescence in colluvial wedges, or we made the simplest likely prediction where no guidance exists. Fortunately, many useful descriptions exist in the literature that can inform predictions (*8*, *17*, *18*). These predictions are that (i) the debris facies have a large residual luminescence due to incomplete sunlight exposure [(*8*, *18*), p. 597] here interpreted as high average luminescence throughout the debris; (ii) the wash facies likely receive extended exposure to sunlight before and during deposition [(*8*, *18*), p. 597]; (iii) luminescence ages, and thus portable luminescence given consistent sensitivity, decrease toward the surface within the finer-grained wash facies due to continuous sedimentation [(*18*), p. 603]; and (iv) luminescence ages should decrease with height in a buried soil up to a previous surface due to sufficient sunlight exposure, the possible influx of aeolian sediment, and pedoturbative reworking [(*1*, *8*), p. 7] (*18*, *19*). We use these observations to develop predictions shown schematically in Fig. 1B. Note that this luminescence prediction is specific to the large aliquots of the portable luminescence method, which measures tens of thousands of grains at a time.

**Fig. 5.**
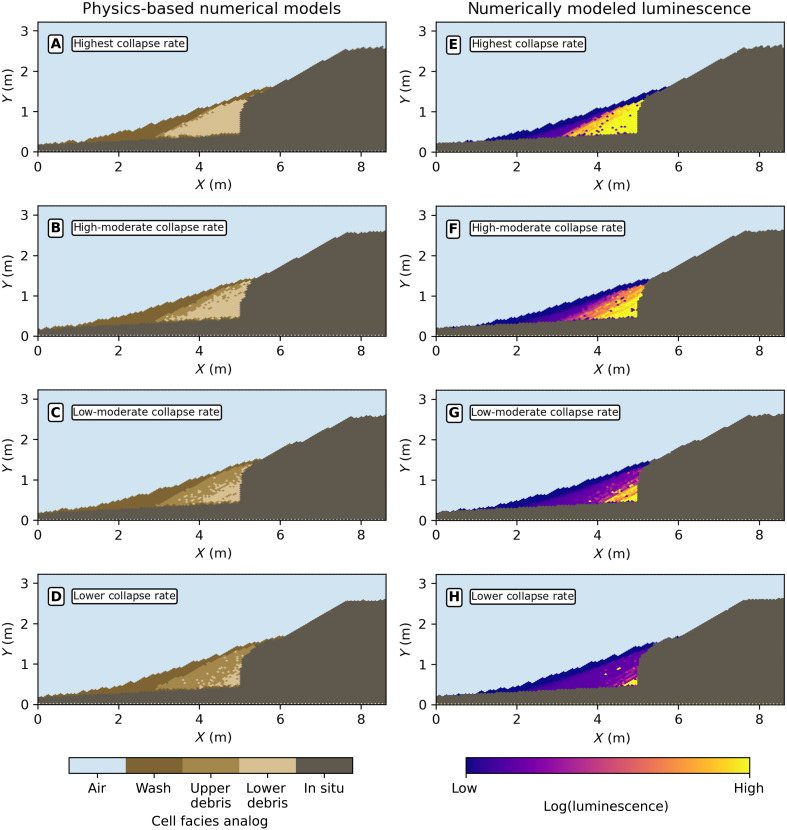
Results of the numerical model and predictions of the spatial distribution of luminescence as a function of changing the lateral collapse rate while holding other constrained parameters constant (e.g., soil turbation rate, scarp height, etc.). Note that the panels on the left side correspond with the panels on the right side [e.g., (A) and (E) are from the same model run]. At high collapse rates relative to turbation rate (**A** and **B**; **E** and **F**), there is significantly less bleaching of luminescence due to the limited amounts of sunlight exposure from the initiation of collapse and burial of cells. At lower collapse rates relative to turbation rate (**C** and **D**; **G** and **H**), bleaching is more extensive largely because of the greater amount of time cells spend exposed to sunlight. In all runs, a small unbleached zone exists within the lowermost of the lower debris facies. Dark spots in (E) and (F) represent cells of low luminescence from the upthrown soil on the footwall and/or sunlight exposure on the fault scarp.

For numerical model predictions of spatially variable luminescence, we adapted continuous-time stochastic cellular automata model (*16*) to include the physics of luminescence. To summarize, the cellular automata model includes mechanics for disturbance-driven (or pedoturbative) mobile regolith transport, mobile regolith production from parent material, and lateral collapse using established geomorphic transport laws (*20*–*23*). Each transport process in the model operates at rates set by parameter values, which can be tuned for a specific field site to simulate a colluvial wedge (Fig. 5). Here, we use parameters constrained for the Deep Creek natural exposure (see the Supplementary Materials for model details) with the only remaining free parameter being the lateral collapse rate, which we explore below. In particular, the lateral collapse process represents destabilization of an individual cell and collapse of it and all cells directly above it in an analog for shallow rotational failure. Cohesive block collapse is not explicitly included because of the limitations of our model. However, we note that colluviation at the field site observed in modern talus slopes of identical lithology did not show cohesive block collapse. For the luminescence physics, we fit our sunlight exposure experimental data and a regression of portable luminescence to equivalent dose from OSL dating (*11*) to general-order kinetic and linear equations, respectively (Fig. 4) (*24*–*26*), and solved for the change in luminescence during transport in the model.

The numerical model predicts that the luminescence of sediment is sensitive to the relative rates of mobile regolith disturbance versus collapse via modulation of the total time of sunlight exposure. Sunlight exposure occurs either while sediment is on or transported along a sunlight exposed surface or while in gravitational free fall during collapse. The model results show that higher collapse rates promote higher luminescence, whereas higher disturbance rates produce lower luminescence (Fig. 5). At the highest collapse rates relative to other parameters such as disturbance rate, the luminescence is high throughout the colluvial wedge. When collapse and disturbance rates are comparable, the luminescence will vary between high and low in an interbedded pattern with a gradient toward lower luminescence at the surface (e.g., Fig. 5G). This modeled interbedding occurs because of stochastic episodes of collapse and sunlight-exposing reworking of collapsed sediment. At the lowest collapse rates, the luminescence is generally very well bleached across the wedge because of the ongoing reworking by disturbance processes such as pedoturbation. Conversely, the luminescence is higher when collapse rates are elevated because of inheritance luminescence of the collapse-derived parent material.

Note the differences and commonalities in the predictions from the conceptual model and the numerical model. First, at the highest collapse rates, the predictions of the numerical model (Fig. 5, A and B) are virtually identical to the conceptual model (Fig. 1B). The predictions differ when the collapse rates are comparable to the fixed mobile regolith disturbance rate calculated for the Deep Creek natural exposure (see Fig. 5, C and D, and the Supplementary Materials). In this part of the parameter space, modeled collapse events occur infrequently enough that the disturbance process, an analog for pedoturbation, can rework the collapse debris and generate complexity in the pattern of luminescence (e.g., Fig. 5C). To analyze whether this complexity is present in nature beyond the predictions of the conceptual model, we turn to a comparison between the model and field data.

### Model and data comparison

Both the conceptual and numerical models accurately predict aspects of the spatial pattern of luminescence observed at the Deep Creek natural exposure. First, the conceptual model appears to predict the first-order features such as a gradient of decreasing luminescence with distance from the fault. Similarly, the numerical model captures the first-order pattern of luminescence and additional details such as the interlayering of high and low luminescence and the spatial variance farther from the fault, particularly model runs with moderate lateral collapse rates relative to disturbance rate (Fig. 5, C and G). These results suggest that the long-standing conceptual model reflects the basic mechanics of colluvial wedge formation and is in general agreement with established sediment transport theory.

However, both the conceptual and numerical models do not account for some details of luminescence at the Deep Creek natural exposure, such as the zones of high/low luminescence or stratigraphic inversions with high luminescence above low luminescence (Figs. 1 and 3). For example, the low luminescence in the lowermost debris facies (which fill the fault fissure; Fig. 2) shows lower luminescence than predicted by either model. In addition, the numerical model appears to underestimate the size of the high/low zones of luminescence in the fault-proximal zone (Figs. 3 and 5).

Before we can interpret these model failures, it is important to consider how luminescence is removed by sunlight in a colluvial wedge environment. First, the time needed for a sediment particle to gravitationally fall the distance of the scarp is insufficient to induce substantial bleaching when more than tens of seconds is needed in direct sunlight (~0.5 s; Fig. 4A). Hence, we expect that sunlight exposure during time in freefall, which could be sensitive to whether transport happens during day or night, is less important of a factor to explain the luminescence patterns.

Instead, sunlight exposure of sediment must primarily occur either on the fault scarp face, in the upper footwall soil before collapse, or following collapse on the surface of the debris pile. Of these options, prior sunlight exposure on the scarp face or upper footwall soil appears less likely to explain our patterns because modeled collapse events appear to mix sediment, producing a more dispersed rather than interbedded pattern (dark spots in Fig. 5, E and F). We note that this is partly a result of our modeling method, which does not include intact block collapse that may need to be considered at other field sites. This reasoning suggests that sunlight exposure of surficial sediment on the hanging wall following/between collapse events is the most likely process for removal of luminescence. Thus, the regions of high luminescence proximal to the fault scarp, i.e., stratigraphic inversions in luminescence, likely show episodic aggradational events that are rapid enough to bury sediment and preclude sunlight exposure, e.g., collapse events. When the stratigraphic inversion in luminescence is accompanied with a change in sedimentary structure such as bedding, it may reflect remobilization and/or erosion of previously deposited wedge material.

The comparison between models, field data, and apparent process time scales suggests a complex and stochastic nature of deposition, erosion, and reworking in the formation of colluvial wedges. First, we note locations where a stratigraphic inversion in luminescence accompanies a change in sedimentology and interpret these as distinct facies changes that represent a change in sediment transport processes. The lower mapped unconformity (location iv in Fig. 3) may represent disturbance, reworking, and/or erosion of collapsed debris material before another episode of rapid aggradation indicated by the overlying luminescence inversion. The luminescence and sedimentology pattern could also be due to a block collapse event. The bedding appears close to the angle of repose observed in nearby talus piles, which complicates distinguishing talus deposition from intact block collapse. In contrast, the uppermost unconformity (location v in Fig. 3) has a more planar contact than the lowermost one but a much greater contrast in luminescence. One possible explanation is that a change in sediment transport drivers, such as climate fluctuations or extreme weather, led to a possible period of reworking or erosion of the top of the wedge, followed by deposition of a new package of colluvium. Alternatively, a later phase of scarp collapse could be possible. We also suggest a possible link between this unconformable zone and a region of higher luminescence in the fault-distal part of the wedge (left of location v in Fig. 3). An alternative possibility is that the upper unconformities and the multiple fault strands represent a second earthquake event with historic timing, although we do not have the data to explore this further here.

Another indicator of the complex interplay of sediment transport processes is the variance in the luminescence in the fault-distal parts of the wedge. The mottled appearance of the luminescence in the Deep Creek natural exposure may be a combination of both provenance and the three-dimensional paths of sediment across the wedge and the stochastic nature of sediment transport. Such variance appears in the results of the numerical model due to stochastic sediment transport and the sunlight exposure history of a sediment cell state, although we do not model three-dimensional transport here (Fig. 5, A to C). A final possibility is the inclusion of aeolian sediment with low luminescence into the wedge stratigraphy creates variation in the luminescence. However, we note that the ratio of infrared to blue stimulated luminescence is constant between the wedge and parent material, suggesting uniform lithology and little to no aeolian signal in our results. While aeolian sediment additions are possible at Deep Creek, they are not needed to qualitatively explain the spatial distribution of luminescence across the wedge.

Our interpretation of stochastic sediment transport is further supported by the similarity of apparent process time scales obtained from the various tracer-delineated units (Table 1). Rapid depositional episodes represented by high luminescence and sedimentary properties within the debris facies and nearest-surface units produce apparent depositional time scales in the range of hundreds of years or less. This time scale is apparently on the same order of magnitude as the overlying wash facies–type units, which are predicted to have slower depositional rates in both the conceptual and numerical models and as indicated by the sedimentology and low luminescence. The matching time scales suggest that both processes that produce debris facies and those that produce wash facies occur synchronously up until the scarp is largely eroded and collapse processes diminish in likelihood. This finding suggests that the conceptual and numerical model’s two-phase style deposition, i.e., that a short time scale phase of rapid gravity-driven collapse followed by long time scale gradual diffusive-like deposition, may oversimplify the actual post-earthquake depositional sequences in colluvial wedges.

### Implications

The foremost implication from this study is that, while the basic transport mechanics of the conceptual model appear largely correct, colluvial wedge formation is a more complex depositional system with components of multiple sediment transport mechanisms, erosion, and reworking. The influence of these depositional and/or erosional geomorphic processes could introduce preservation bias effects that have yet to be fully explored. First, we note that our results show that wedge-forming and wedge-modifying processes act concurrently. The processes associated with the debris and wash facies both occur at the same time scales and can produce different luminescence spatial patterns depending on transport process. This finding agrees with long hypothesized debris and wash formation mechanisms in the literature (*3*), and here, we present longer–than–modern–time scale evidence of this behavior. However, our results justify greater emphasis on the stochasticity and concurrency of various processes, such as pedoturbation, gravitational collapse, and other site-specific factors, to explain the distribution of luminescence seen at the Deep Creek natural exposure and in colluvial wedge morphostratigraphy more broadly.

Next, both the conceptual and numerical models underestimate the extent to which rapid aggradation episodes are interspersed with periods of low aggradation, hiatus, and/or erosion. These episodes are reproduced in the numerical model but are considerably smaller in duration and deposited volume than the evidence from the Deep Creek natural exposure luminescence map (Fig. 3). The underestimation in the numerical model may arise because the modeled collapse process has a time-independent (constant) probability of collapse per unit time and a limited amount of collapse-able material (e.g., no block collapse) due to the limitations of our cellular automata. No value of collapse in the numerical model can fully reproduce the Deep Creek natural exposure’s luminescence pattern, particularly the basal zone (Fig. 3) beyond a qualitative sense (Fig. 5). This likely indicates that real-world collapse occurs in a larger, less frequent, and at a time-dependent rate. An example could be that nonseismogenic collapse of fault scarps tends to occur during extreme precipitation events wherein raised pore water pressure promotes mass failure. Another possibility is that parent material cohesion changes over time as result of pedogenic processes such as carbonate precipitation, clay infiltration, and/or case hardening.

The observation of deposition dynamics beyond those predicted from the models also appears in the apparent process time scales extracted from geochronology (Table 1). Critically, it appears that the apparent time scales of the debris aggradation are similar in order of magnitude with the wash aggradation episodes. Furthermore, there is overlap with the apparent time scales of the formation of soil properties. This brings up challenging possibilities: If collapse occurs between intervals long enough to allow for soil development, could it be possible to create the patterns assumed in the colluvial wedge conceptual model in a nonseismogenic manner? Put otherwise, would an investigator interpret more seismogenic events than are truly recorded in colluvial wedge stratigraphy? Note that soil formation rates vary with time and environment. At a field site not far from the Deep Creek natural exposure, soil formation rates on scarp colluvium varied greatly over time with an enhanced rate occurring between 8 and 13 ka (*27*). If an earthquake occurred during a period when soil formation is fast relative to collapse time scales, then it may be possible that multiple conceptual model style depositional sequences could result from one earthquake event. If this scenario is interpreted as multiple earthquake events, then there is a possibility that the seismic hazard could be mischaracterized.

Alternatively, could stochastic deposition and erosion mask earthquake events that are closely spaced in time relative to site-specific colluvial wedge time scales? There is recognition that geomorphic processes modify tectonic features over time in ways that affect seismic assessments (*1*, *28*–*32*) and that stochastic sediment transport can produce preservation bias effects that greatly complicate interpretations of climatic or tectonic change (*33*). If our luminescence-informed interpretations are correct, then postseismic episodes of deposition, erosion, and modification that are hard to detect from sedimentology alone are possible. One example could be bioturbation mixing seismogenic sediment with nonseismogenic sediment or potentially even removing seismogenic sediment before complete degradation of the fault scarp. Such modification could bias geochronology toward apparently younger ages by introducing modern carbon or sunlight-exposed sand without providing unambiguous field evidence to paleoseismologists. However, note that modern standards of paleoseismic practice control for this by rating the quality of the evidence, for a given event, such as tectonic deformation of a prior colluvial wedge or pedogenic features, to define multiple seismic events from colluvial wedge deposits (*34*). In cases where event interpretations are ranked by supporting evidence, there are approximately 15 to 20% events that have weaker evidence that propagates uncertainty into recurrence interval calculations (*2*).

The effect of apparent process time scales on earthquake preservation likely depends on the relative time scale of recurrence interval. Note that the hundreds of years process time scales overlap the recurrence of some earthquake events observed on the Wasatch Fault (*35*) but appear shorter than the thousand-year time scales of average earthquake recurrence (*12*). Consequently, closely spaced earthquake events with hundred-year recurrence could be more susceptible to ambiguous preservation than longer intervals due to possible interbedding of seismogenic and nonseismogenic debris. Recurrence intervals that are much longer may not suffer this challenge. However, regions where recurrence intervals overlap with process time scales may experience a filtering effect wherein relatively short recurrence events are ambiguously preserved compared to long recurrence events. Such preservation effects have a direct effect on recurrence calculations (*2*). Note that such concepts have been long hypothesized (*1*), but here, we present evidence framing the geomorphic conditions that could produce this behavior.

As shown here, luminescence sediment tracing has the potential to benefit interpretations of depositional mechanics in paleoseismic settings using principles similar to those used here. Developing a luminescence map could feasibly reveal depositional episodes that can be compared with suspected earthquake chronologies. Variations in luminescence could also be useful in cases where stratigraphic contacts or bedding is obtuse or ambiguous. For example, an infilled burrow in homogeneous sediment may translocate more or less sunlight-exposed material than a strike-slip altered zone, despite both having a highly disturbed appearance. However, such an interpretation must also reconcile any sedimentological and stratigraphic information available. The luminescence may not be diagnostic on its own as many environmental factors can alter the amount of luminescence beyond sunlight exposure such as mineralogy and local background radiation (*7*, *36*). These factors may need to be considered in more geomorphically complex environments than the field site presented here. However, this new method appears promising as a tool to assist paleoseismology and seismic hazard assessments (*11*).

To conclude, the presence of stochastic geomorphic processes justifies further study on the completeness of the tectonic history of any given fault scarp stratigraphy (*1*, *28*, *30*). Potential preservation bias phenomena have a direct effect on calculations of earthquake recurrence and seismic hazard assessments (*2*). However, addressing these issues may provide insight toward some of the largest questions in seismology such as resolving paleoseismic versus historic records and periodic versus Poissonian earthquake behavior (*2*, *37*) and/or debate between characteristic versus Gutenberg-Richter event distributions (*38*, *39*). Although this study focuses on normal displacement, similar concepts may be applicable to strike-slip and/or reverse fault paleoseismology. Tracer studies, including the methods presented here, may provide a means toward better understanding fault zone sediment mechanics and help address such broader-scale questions.

## MATERIALS AND METHODS

### Cellular automata simulation of the Deep Creek natural exposure

Our simulation of the colluvial wedge present at the Deep Creek natural exposure is built from the “GrainHill” continuous-time stochastic cellular automata model (*20*, *21*). To summarize, a continuous-time stochastic cellular automata model consists of a grid of cells with various states the cell can be in. In GrainHill, the states are “air,” “mobile regolith,” or “in situ parent material.” Cells can transition between states by various processes at rates governed by probability distributions. For example, an in situ parent material cell can transition to a mobile regolith cell following a “weathering” transition function (fig. S1). Furthermore, GrainHill simulates the movement of sediment in geomorphic environments by including transition functions for (i) the weathering of parent material into mobile regolith; (ii) the mobilization and transport of mobile regolith by disturbance, e.g., bioturbation; and (iii) momentum and the rebounding due to elastic collision leading to granular dynamics. Time progresses continuously within the model with cell state transitions occurring in an order set by values from the probability distributions underlying each process. The transition processes of GrainHill and those used in our paper are shown schematically in fig. S1. We add in an additional transition function to simulate the lateral collapse of vertical faces such as a fault scarp (fig. S1A). The resulting model can produce accumulations of mobile regolith cells at the base of simulate fault scarps that resemble colluvial wedge morphology and stratigraphy (fig. S1, B to D).

To simulate the colluvial wedge present at the Deep Creek natural exposure, we apply the cellular automata model using literature values appropriate for the field site. We used a scarp height of 2.0 m, a fault scarp dip of 90°, and a simulation run time of 1000 years based on paleoseismic studies of the Deep Creek natural exposure (*8*, *9*, *11*, *40*). We used a cell size of 5 cm based on the approximate max cobble size, spacing of portable luminescence measurements, apparent thickness of mobile regolith, and computational costs. Cell size does not appear to have a sizeable enough effect on the GrainHill numerical modeling to affect our conclusions (fig. S7) (*16*). We used a mobile regolith disturbance rate using the global-scale regression between hillslope diffusivity and mean annual precipitation (*41*). Using the mean annual precipitation for nearby Levan UT (36 cm/year), we obtain a diffusivity of 3.7 × 10^−3^ m^2^/year, which, in turn, results in a disturbance rate value of ~0.025 cells/year using the conversion formula in the study of Tucker *et al.* (*22*). Note that Hylland (*40*) obtain a scarp diffusivity of 0.52 m^2^/year for the Deep Creek natural exposure, but this value subsumes the lateral collapse of the fault scarp in addition to the diffusivity-like constant and so does not solely capture the “activity” parameter (*42*) needed for the GrainHill framework. For the transition function that converts in situ parent material into mobile regolith, we use a globally averaged value of 1.1 × 10^−5^ m^2^/year using a regression from the arid/semiarid climate data (*43*) and our cell size (5 cm) for the depth. As with the disturbance rate, we convert to units of cells per year using conversion equations of Tucker *et al.* (*22*). We use a value of 25% for the elastic collision factor from GrainHill’s source code (*22*) while noting that this value does play a major change in results (*16*). The values for luminescence growth and decay (κ = 72,732.5; β = 7.538) are obtained from experiments described below. The lateral collapse rate is an unconstrained parameter explored via parameter space exploration with the results described in the following sections. The simulated colluvial wedges are shown in fig. S2.

### Sediment cell classification

To provide a means of comparison between the numerical model and the conceptual model, we classify the modeled sediment cells into analogs for the sedimentogical facies of the conceptual model. To do this, one needs to consider that sediment transport in a model and in reality involves both spatial and temporal components. To analyze these components, we produce scatterplots comparing (i) the ratio of the total vertical displacement versus horizontal displacement of a sediment cell state (∆*y*/∆*x*; a proxy for provenance), (ii) the total linear distance a cell state is transport to, (iii) the time spent undergoing transport, and (iv) the average velocity of a sediment cell state between the time of initial mobilization and time of final deposition. We do this for each numerical model run (figs. S2 to S4).

The points on the scatterplot appear to cluster into groups, which we have classified using cutoff values for velocity. Sediment cell states with average transport velocity greater than 10^5^ m/year are classified as analogs for the debris facies; sediment cells with less velocity than the debris classified cells but higher than 1 m/year are classified as “sorted” as shorthand for the sorted debris facies; sediment cell states with lower velocities than 1.0 m/year are classified as analogs for the wash facies.

We chose these boundaries heuristically from observation of the scatterplots as theory does not yet exist of what the exact value of the boundaries should be. For context, sediment cells gravitationally fall at a velocity of 10^7^ m/year, so the velocity of the debris facies cells subsumes both gravitational freefall and a minor component of slower transport due to elastic collision with the ground, small ledges on the fault scarp, with other falling sediment cells, and some limited disturbance. In contrast, the sorted facies cells contain a larger proportion of overall slower transport either traveling down an angle-of-repose slope (here 30° due to the hexagonal shape of the cells) created by the debris at the base of the scarp or transport due to the mobile regolith disturbance process. Last, the wash facies cells undergo transport almost entirely by the mobile regolith disturbance process and hence have very slow average transport velocities. Note that this cellular automata model does not capture grain shape or size effects so inferences must be made on transport histories instead of sedimentological character.

### Portable luminescence measurements and modeling

To simulate and predict the spatial distribution of luminescence across a colluvial wedge, we added in a luminescence property to the mobile regolith cells and calculated the change due to time of sunlight exposure and background ionizing radiation [conceptually following the study of Brown (*26*)]. For both luminescence growth and decay, we use the data from a sunlight exposure experiment and age regression (*11*). For luminescence growth, we simulate a dose-response curve by regressing portable luminescence measurement versus equivalent dose (Fig. 3B). We found that a linear relationship explains the data (adjusted *R*^2^ = 0.95; *P* = 1.48 · 10^−7^) and used an approximate form to model the growth in the model. However, a 1-ka time span is a fairly short period of time for a luminescence geochronometer, and the growth has a barely noticeable effect on the model predictions compared to the decay due to sunlight exposure. For modeling the luminescence decay, we fit a general-order kinetics model (*24*) to represent the complex collective emissions versus time of electrons undergoing recombination within a multigrain and multimineral sample. The general-order kinetic equation is given as

$$\frac{\mathrm{dL}}{\mathrm{dt}}=-\kappa L^{\beta }$$ (1)

where *L* is the portable luminescence in photon counts, *t* is the light exposure time either in seconds or years for the model, κ is a constant describing the loss rate of luminescence versus the light exposure time, and β is a constant describing the decay order that is analogous to the number of energy transitions an electron undergoes until the final recombination. Eq. 1 solves to

$$L(t)=L_{0}{\left[(\beta -1)\kappa t+1\right]}^{\beta /(\beta -1)}$$ (2)

where *L*_0_ is an initial luminescence intensity at *t* = 0. Eq. 2 is used in the model using values for κ and β from fitting bleaching experimental data (Fig. 3A) (*11*). The κ and β values used in the model are those resulting from fitting all the blue stimulated bleaching data from the three samples and represent an average behavior of portable luminescence with sunlight exposure time (Fig. 3A). For simplicity, we normalized the bleaching experiment luminescence between 0 (fully bleached) and 1 (unbleached sample) and did the same with the numerical model. As we are describing spatial patterns instead of exact values, this normalization is adequate for our purposes.
