# Supplementary material for: Luminescence sediment tracing reveals the complex dynamics of colluvial wedge formation
Source: Sci Adv. 2022 Jun 1;8(22):eabo0747. doi: 10.1126/sciadv.abo0747 (PMC9159699; doi:10.1126/sciadv.abo0747)
Supplement: Supplementary file 1 — Figs. S1 to S8 [file sciadv.abo0747_sm.pdf]

Science Advances
AAAS

Supplementary Materials for

Luminescence sediment tracing reveals the complex dynamics of colluvial wedge formation

Harrison Gray *et al.*

Corresponding author: Harrison Gray, hgray@usgs.gov

Sci. Adv. **8**, eabo0747 (2022)
DOI: 10.1126/sciadv.abo0747

This PDF file includes:

Figs. S1 to S8

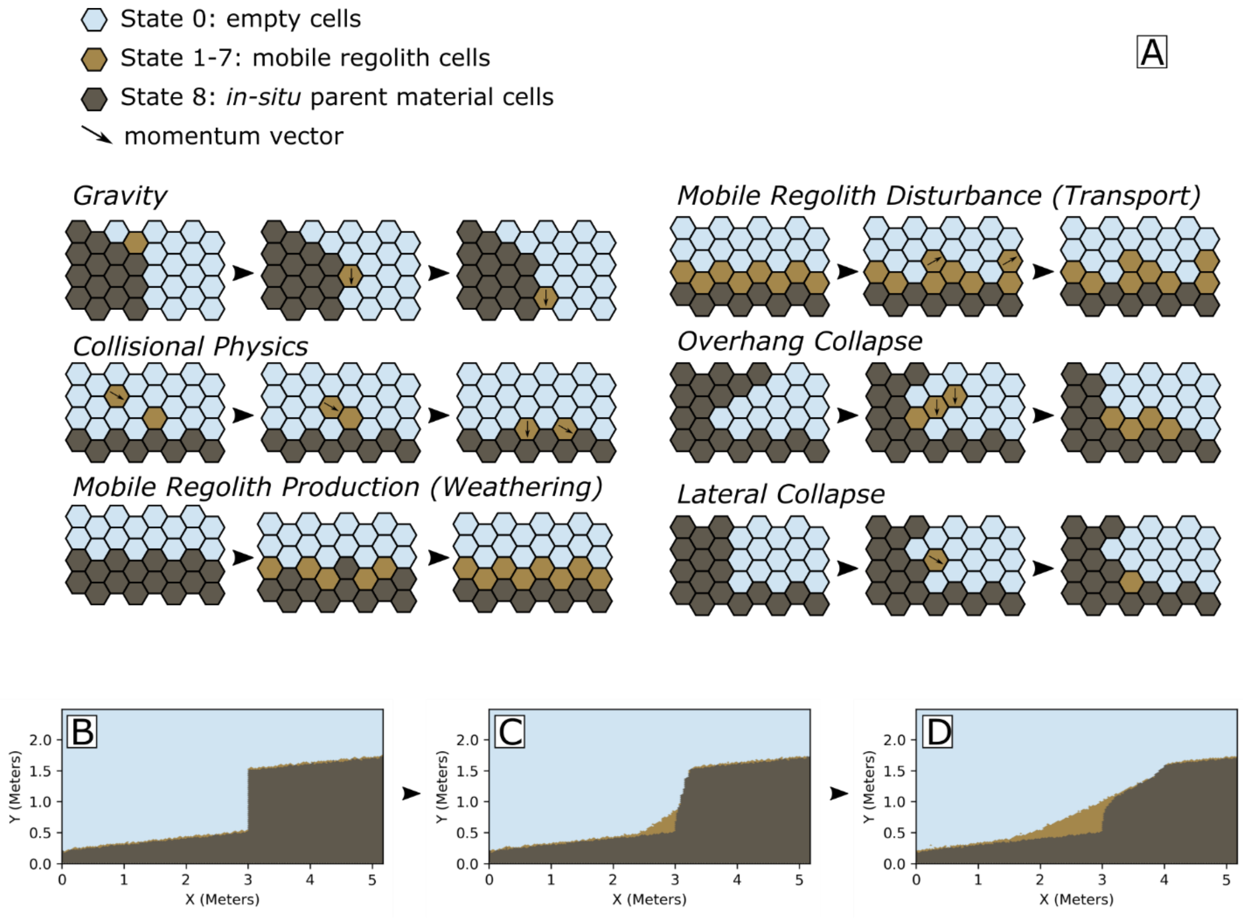

Figure S1.

Example of the “GrainHill” continuous-time-stochastic cellular automata model using in this study. A) Cell states: “air” represents a cell state with no tracked property (such as luminescence); “mobile regolith” represents sedimentary material either at rest or in transport with a tracked property such as luminescence; “in-situ” cells represent a cell state that has not undergone yet transport by the mobile regolith production or collapse processes. B) Initial condition of the numerical model representing a vertical fault scarp. C) Immediately after model start, the relatively rapid collapse process is dominant and produces a pile of mobile regolith cells at the base of the scarp. D) Over time, the additional transition processes act to produce a larger colluvial wedge form. Use of the parameters specific to the field site produce colluvial wedge forms presented in Figure S2.

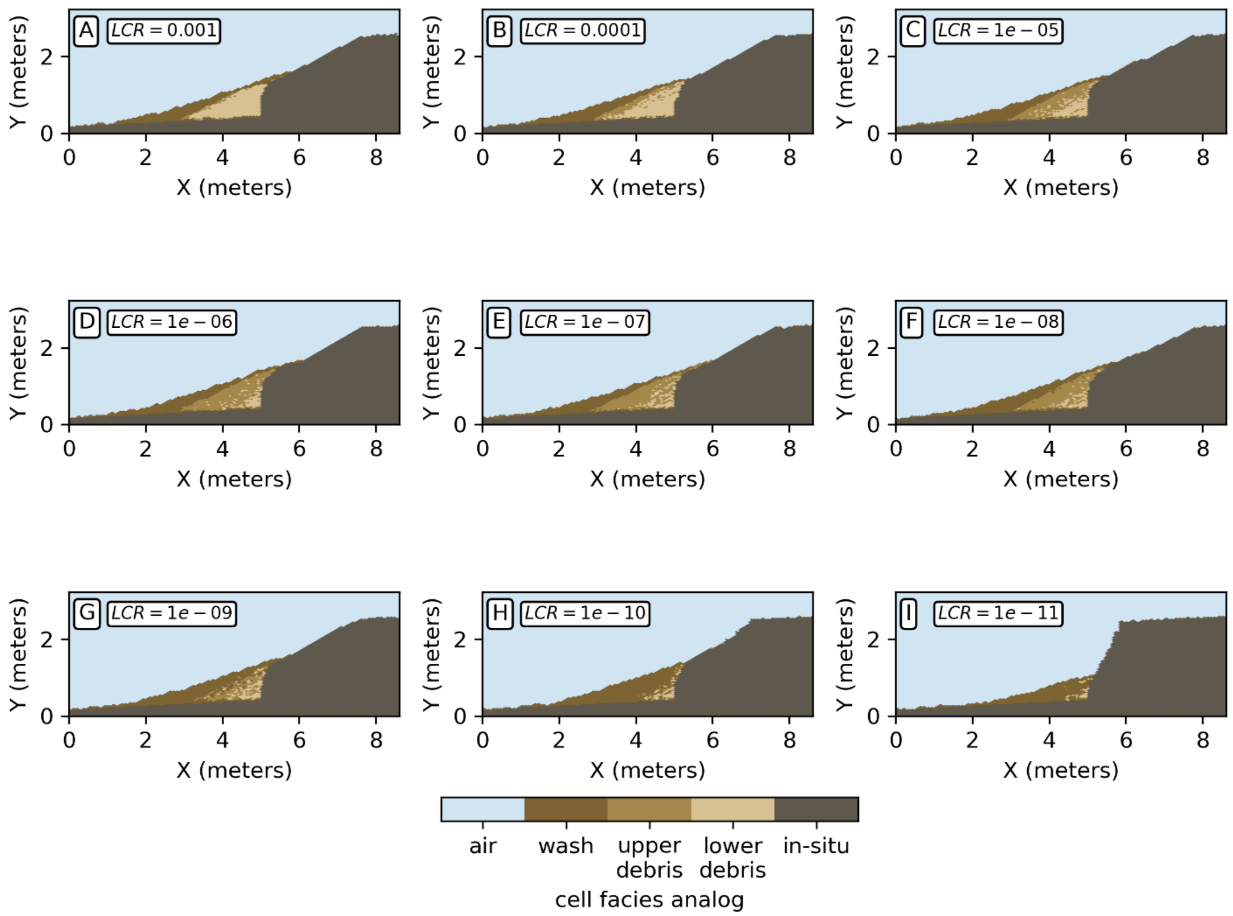

Figure S2.

Results of the numerical model showing modeled colluvial wedge morphology and resulting cell facies analogs. Cells are classified into analogs for the various facies in the colluvial wedge conceptual model based on the characteristics of their transport histories (Fig S3). A-I) Effect of varying the sole unconstrained parameter, the rate for the lateral collapse transition process. The LCR value is the fraction of the rate of gravitational fall; an LCR of 1 means that the in-situ cells collapse at the rate of gravity, such as entirely unconsolidated material. Here, we vary the value from LCR = 0.001 (essentially immediate collapse of the fault scarp) to 10^{-11} which, after 1000 years of model time, results in a steeper scarp than observed at the DCNE field site and is likely an underestimate of the real collapse rate.

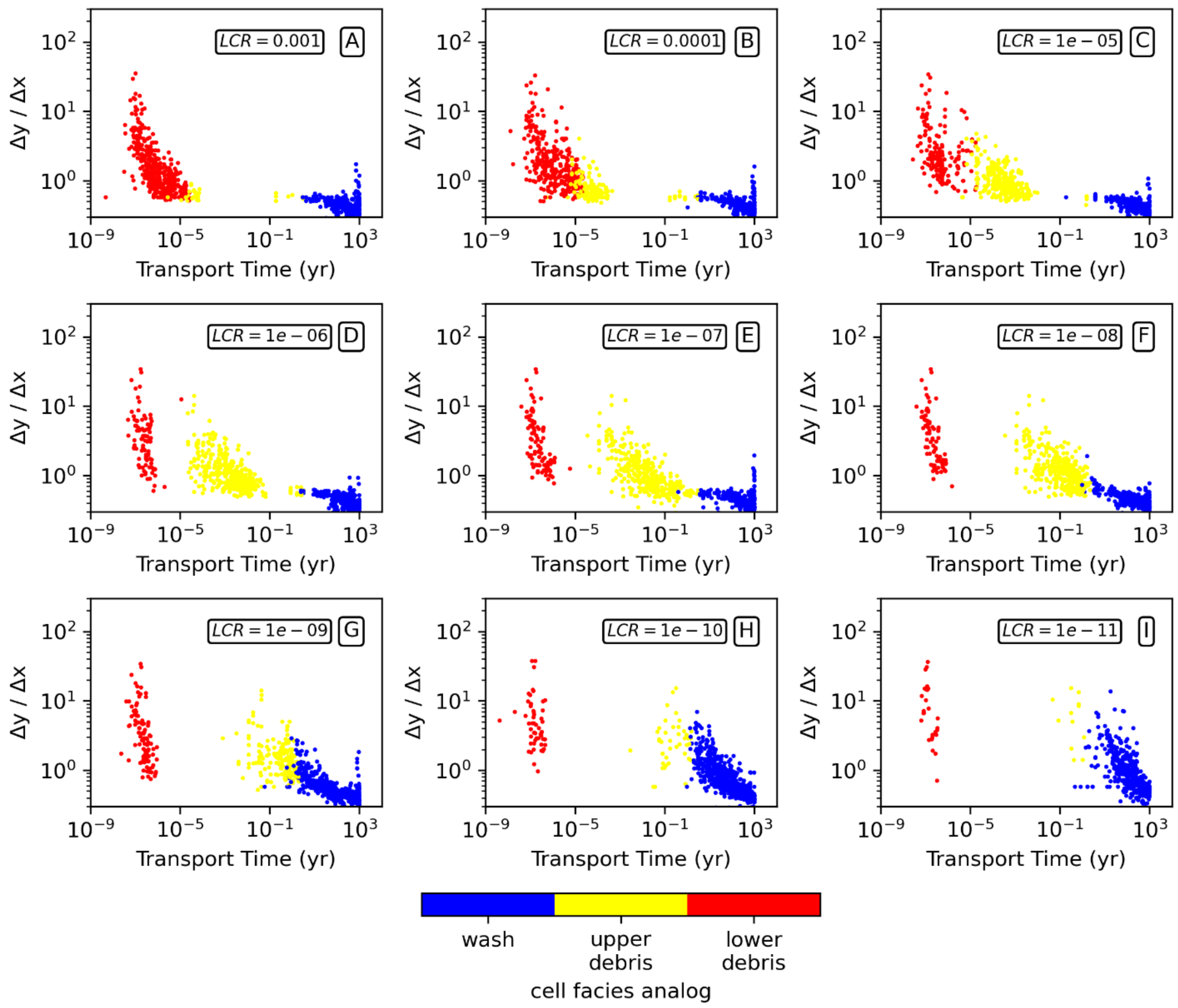

Figure S3.

A-I) Scatter plots of the total time spent by a sediment cell state from initial mobilization to final deposition versus $\Delta y / \Delta x$, where Δx and Δy are the total horizontal and vertical displacements over the model run. Higher values of this ratio indicate a greater vertical distance of transport versus horizontal transport. For example, a high value indicates a cell falling from the top of the scarp to the debris pile, whereas a low value indicates transport across a wide region such as across the surface of the colluvial wedge. In these plots, we observe clustering of cells and classify these clusters based on the transport velocity (Figure S4) into analogs for sedimentary facies in the colluvial wedge conceptual model. Colors indicate interpreted cell facies analog. In high collapse rate model runs (A) most of the sediment cells fall into either the lower debris and wash facies. For lower collapse rates (B-I), the greater relative role of reworking (mobile regolith disturbance, Figure S1) causes a greater proportion of cells falling into the lower debris or wash facies.

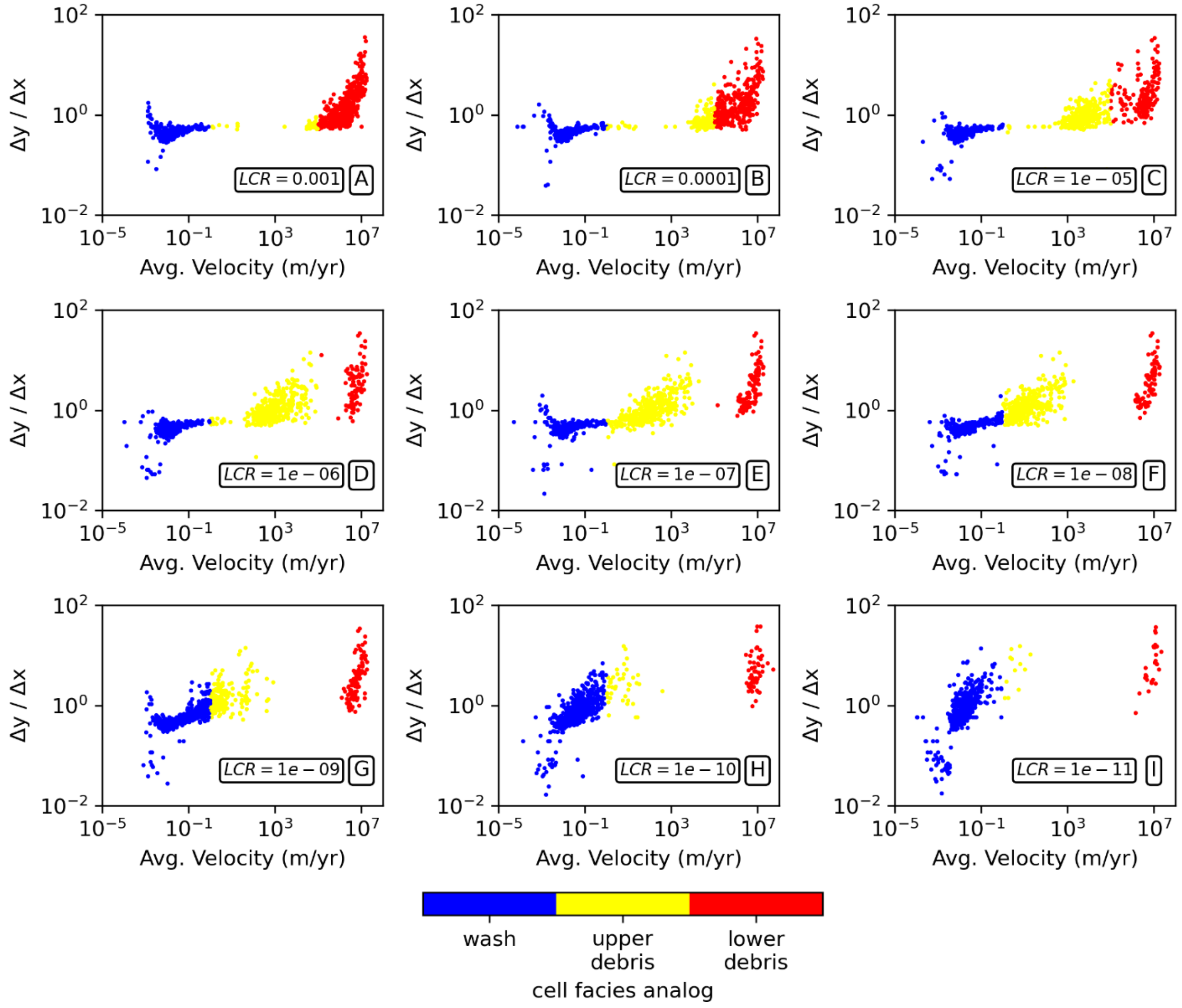

Figure S4.

A-I) Scatter plots of the average velocity while a sediment cell is in transport, versus the ratio of $\Delta y / \Delta x$. Velocity is calculated by dividing the linear distance of transport ($\sqrt{\Delta y^2 + \Delta x^2}$) by the total time of transport. As with Figure S3, we observe clustering of mobile regolith cell transport histories. We find that mobile regolith cells with average transport velocities greater than 100 meters per year form a persistent cluster resembling the lower debris facies; cells with velocities lower than 1 meter per year appear to cluster into an analog for the wash facies; finally, cells between these velocities appear to form an analog for the upper debris facies.

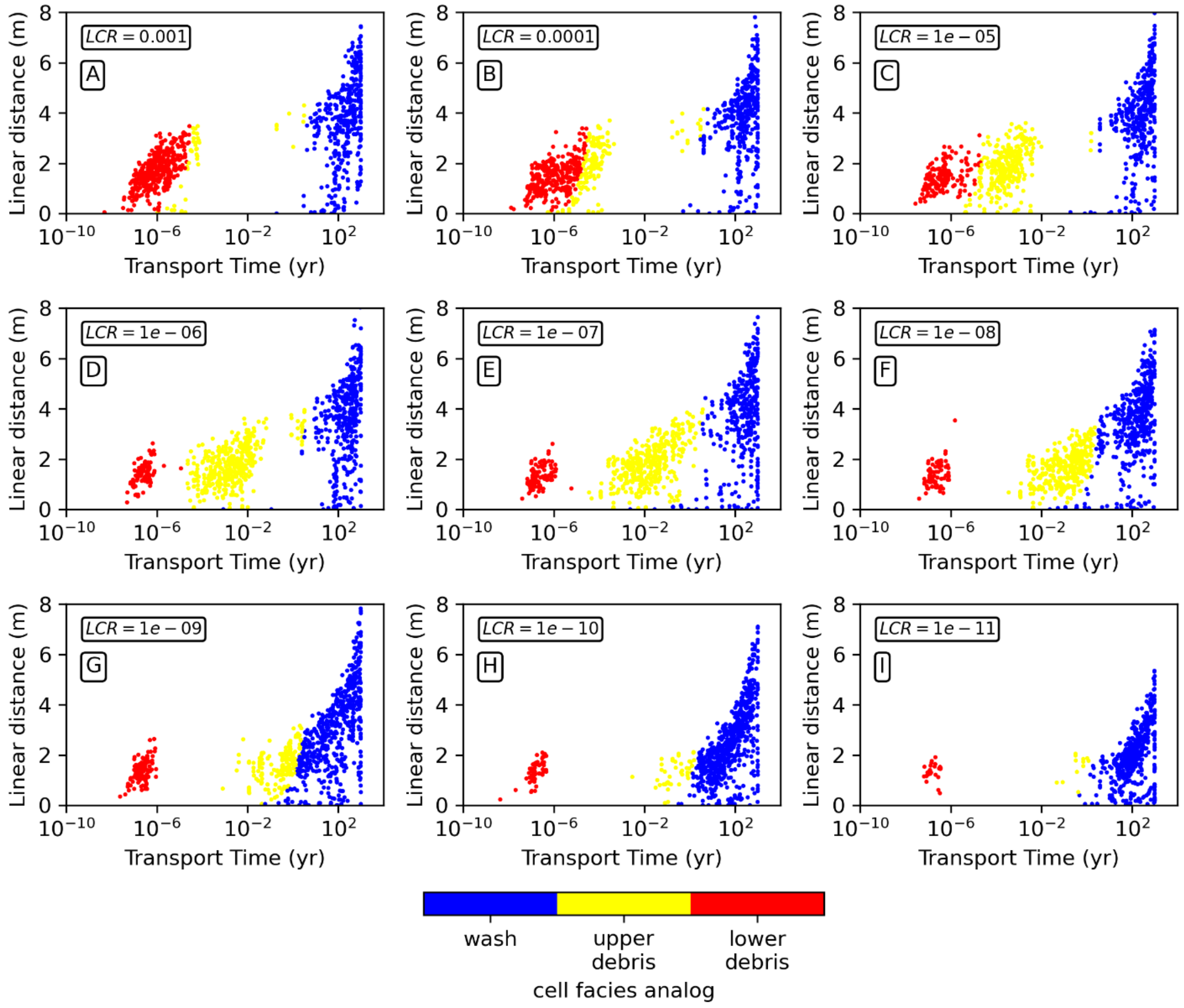

Figure S5.

A-I) Scatter plots of the time spent in transport versus the linear distance of transport. The longer a cell spends in transport, the further it travels and the slower its overall velocity. As with Figure S3 and Figure S4, we observe clustering of cell transport histories and use this observation to classify cells into analogs for sedimentary facies.

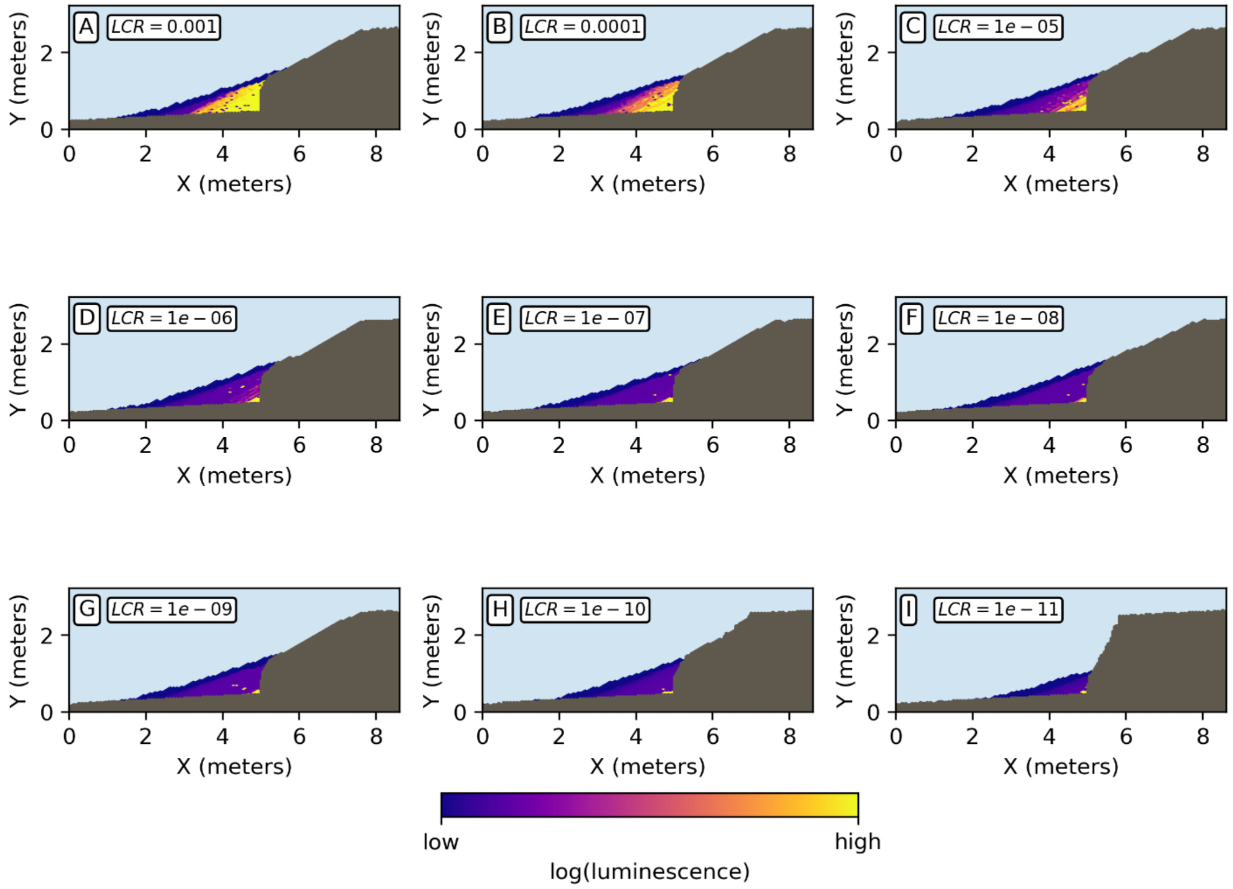

Figure S6. A-I) Results of the numerical model and predictions of the spatial distribution of luminescence as a function of changing the lateral collapse rate. At high collapse rates (A-C), there is significantly less bleaching of luminescence due to the limited amounts of sunlight exposure from the initiation of collapse and burial of cells. At lower collapse rates (D-I), bleaching is more extensive largely due to the greater amount of time cells spend exposed to sunlight after collapse. In all runs, a small unbleached zone exists within the lowermost of the lower debris facies. Dark spots in (A-C) represent cells of low luminescence from the upthrown soil on the footwall and/or sunlight exposure on the fault scarp.

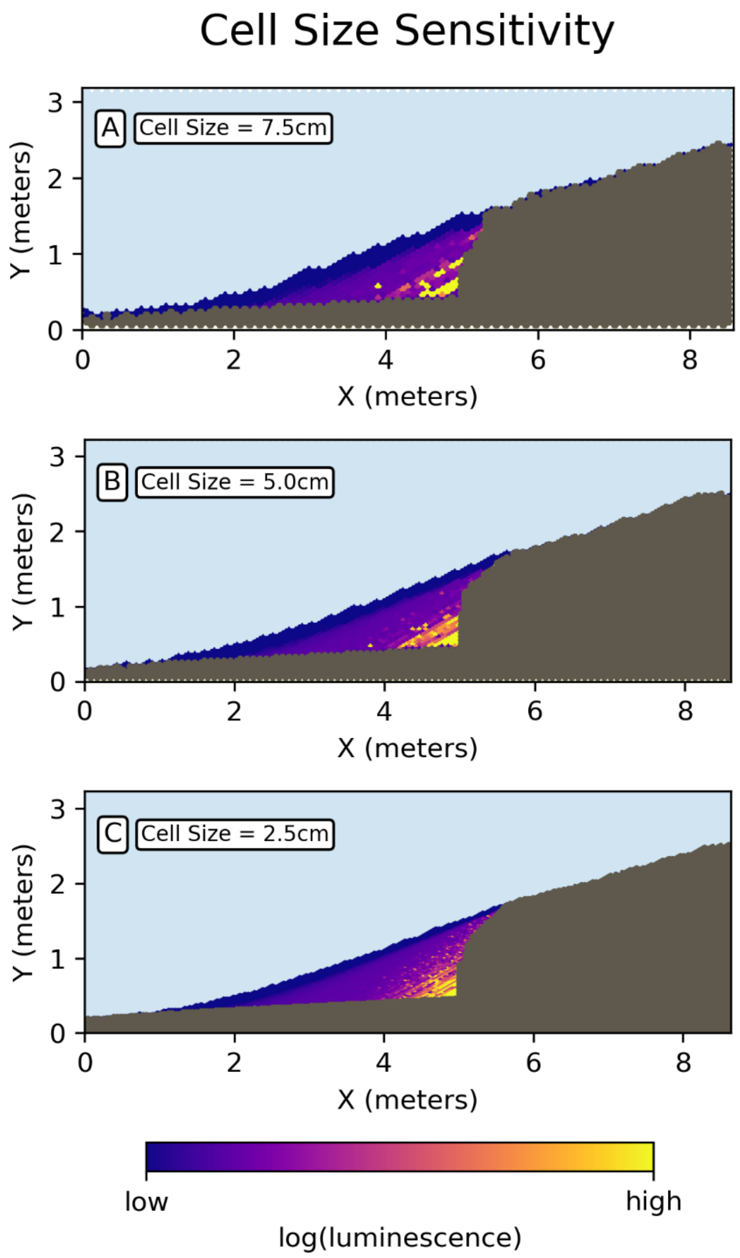

Figure S7. A-C) Results of the numerical model with LCR = 1.0e-5 when varying the cell size from 2.5cm, to 5cm, to 7.5cm. All cell size model runs are qualitatively similar with lower cell sizes showing higher resolution. We use a cell size of 5cm for the main results of the paper due to a balance between typical clast size at the field site, resolution, and model run time. The cell size does not change the conclusions of our numerical modeling results because the model is primarily to show that a simplified numerical version of the conceptual model does not capture the complexity of the luminescence sediment tracer field data.

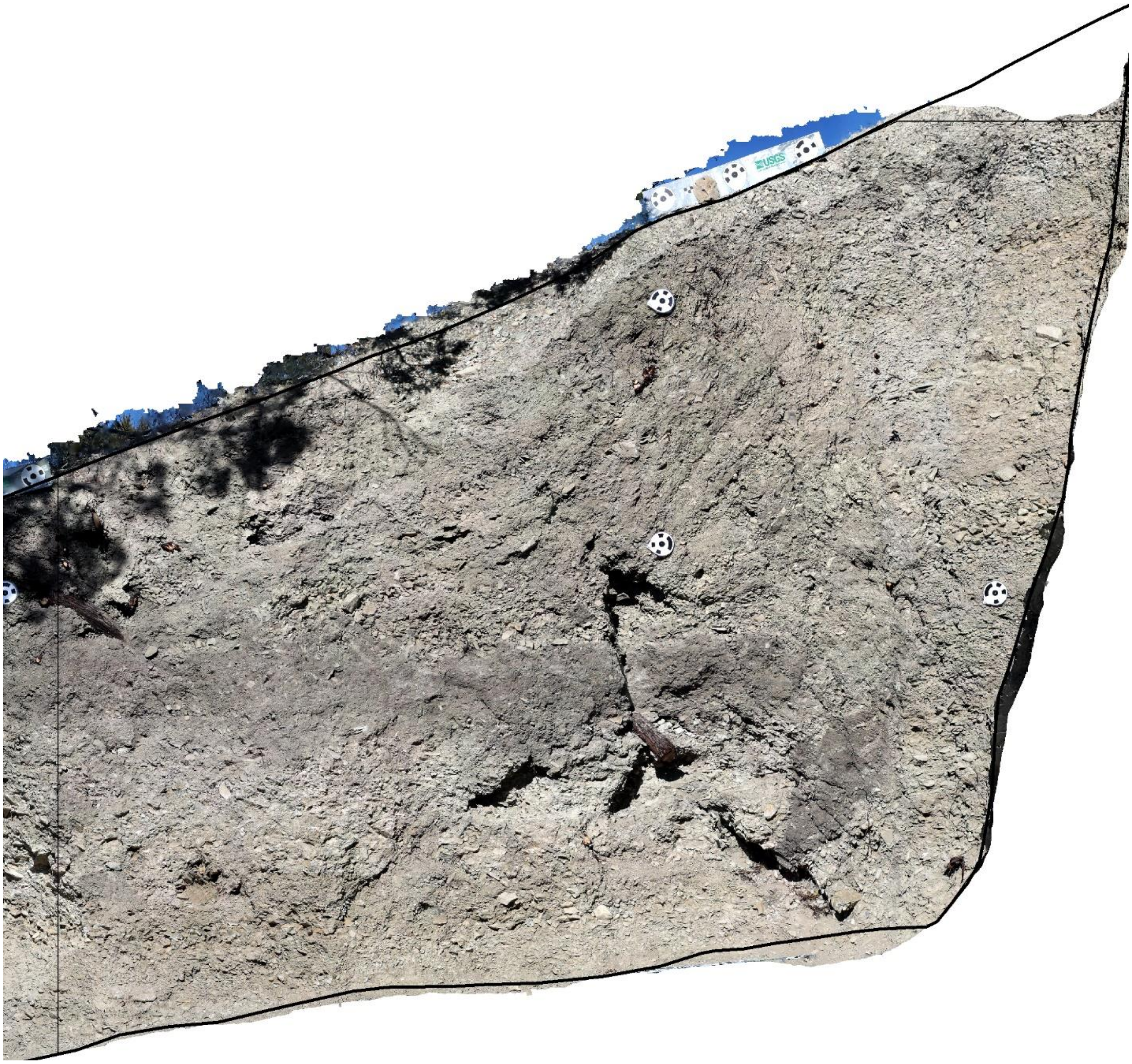

Figure S8.

Orthophoto of the Deep Creek Natural Exposure without annotations or measurements. Photogrammetry bar is 40 cm long.
